# Luminescent Nanoparticles of Gd_2_O_3_:Eu^3+^ Encapsulated Within SiO_2_–PMMA Gel–Polymer Hybrid Matrix: Synthesis and Optical Properties

**DOI:** 10.3390/gels12060546

**Published:** 2026-06-18

**Authors:** Martin Rodolfo Palomino Merino, Juan de la Cruz Quiroga, Oliver Isac Ruiz Hernández, Oscar Mario Martínez Bravo, Benito de Celis Alonso, Angélica Gutiérrez Franco, Miller Toledo Solano, Claudia Mendoza Barrera, Humberto Salazar Ibargüen

**Affiliations:** 1 Facultad de Ciencias Físico Matemáticas, Benemérita Universidad Autónoma de Puebla, Avenida San Claudio y 18 Sur, Colonia San Manuel, Ciudad Universitaria, Puebla 72570, Mexico; juandlcruz0@gmail.com (J.d.l.C.Q.); oliverisac@yandex.com (O.I.R.H.); omartin@fcfm.buap.mx (O.M.M.B.); bdca@fcfm.buap.mx (B.d.C.A.); col538401@colaborador.buap.mx (A.G.F.); mtoledoso@conacyt.mx (M.T.S.); cmendoza@fcfm.buap.mx (C.M.B.);; 2 Centro Interdisciplinario de Investigación y Enseñanza de la Ciencia (CIIEC), Benemérita Universidad Autónoma de Puebla, Puebla 72570, Mexico

**Keywords:** sol–gel processes, gel–polymer matrix, hybrid materials, luminescence

## Abstract

Luminescent gadolinium oxide nanoparticles doped with europium were synthesized through a precipitation reaction using gadolinium and europium nitrates as precursors. The europium-doped gadolinium oxide nanoparticles were incorporated first into a gel matrix of silicon dioxide and second by mixing with polymethyl methacrylate. Both processes are synthesized by the simultaneous hydrolysis of tetraethyl orthosilicate and polymerization of 3-(Trimethoxysilyl) propyl methacrylate. The solid samples obtained are round in shape with a size of about 2.5 cm, which makes the material easy to handle to test different applications. The inclusion of Gd_2_O_3_:Eu^3+^ nanoparticles increases the level of absorbance in the ultraviolet region, which allows for the improved emission of the material at a wavelength of around 610 nm. Furthermore, it enables easy doping of the material and the fabrication of thin films and monoliths with potential optical applications.

## 1. Introduction

Gadolinium oxide (Gd_2_O_3_) has gained significant interest due to its unique properties, making it useful in several technological applications. It exhibits high transmission in the visible region of the electromagnetic spectrum, a wide bandgap (5.8–6.4 eV), low phonon energy (~600 cm^−1^), outstanding chemical stability, and a high neutron absorption cross-section [1,2]. These attributes make it useful in semiconductor devices such as dye-sensitized solar cells [3], as a phosphor with attractive properties for optoelectronic applications [4], and for neutron detection and capture [5,6]. Nanoparticles of Gd_2_O_3_ possess unique size-dependent properties, and their high surface area allows their use for a wide range of applications, including catalysis and molecular sensing [7,8]. Moreover, Gd_2_O_3_ nanoparticles showcase high biocompatibility [9,10,11], leading to their remarkable use as contrast agents in MRI scanning in the medical field [12]. Rare-earth-doped Gd_2_O_3_ nanoparticles have also demonstrated promising luminescent properties [12,13,14,15].

Hybrid materials and nanocomposites have multiple components bonded at the nanometric or molecular level. Technological research has shown that specific properties can be achieved with these materials that otherwise could not be possible with a single component. These hybrid materials often exhibit improved mechanical properties over their individual components [16,17]. Additionally, several properties, such as optical [18] and electrical [19] characteristics, can be fine-tuned by changing the ratios and compositions of the components. Notably, particular phosphors retain their luminescent properties while gaining photostability when incorporated into these materials [20,21]. In particular, PMMA–SiO_2_ hybrid films have shown tunable optical, electrical and structural properties by changing the coupling agent concentration, making it a promising material for next generation electronic devices [22,23]. On the other hand, the doping of silica-polymer hybrid materials with some luminescent materials can keep or enhance their photoluminescence activity [24,25].

In this work luminescent europium-doped Gd_2_O_3_ (Gd_2_O_3_:Eu^3+^) nanoparticles were incorporated into a silica-polymethyl methacrylate (SiO_2_–PMMA) hybrid matrix, which was synthesized by sol-gel method. The Gd_2_O_3_:Eu^3+^ nanoparticles served as phosphors [26] while the SiO_2_–PMMA provided the mechanical properties to the hybrid material. The resulting luminescent composite can be efficiently used in solid state applications such as light emitting diodes (LEDs) or thin film, with tunable properties as change in color by adding different dopants as in some reported works [27,28]. The optical properties of the composite were analyzed using UV–Vis and photoluminescence spectroscopies. The morphology of the composite was observed through scanning electron microscopy (SEM) and atomic force microscopy (AFM) while its chemical structure was assessed using Raman spectroscopy. Then, the Gd_2_O_3_:Eu^3+^ nanoparticles and the SiO_2_–PMMA hybrid matrix were characterized.

## 2. Results and Discussion

**Figure 1:**
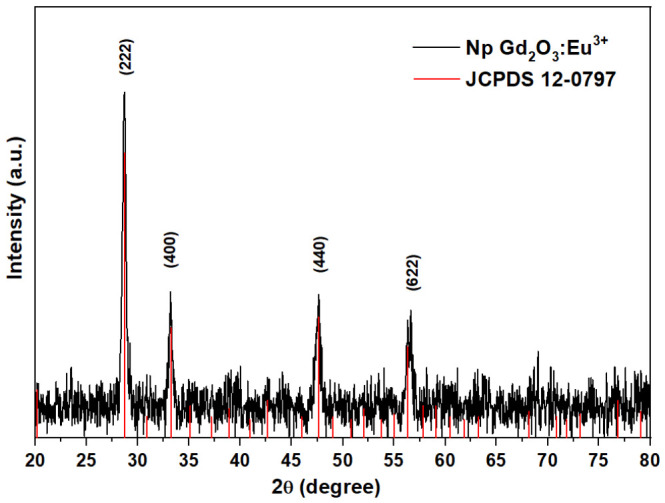
XRD spectra of the Gd_2_O_3_:Eu^3+^ nanoparticles.

The analysis of the XRD data, presented in Figure 1, confirms that the Gd_2_O_3_:Eu^3+^ particles have a crystal structure of Gd_2_O_3_ in the cubic phase after heat treatment [29]. The diffraction peaks corresponding to the (211), (222), (400), (431), (440), and (622) planes are located at 2θ values of 20.12°, 28.69°, 33.18°, 42.64°, 47.65°, and 56.30°. Due to the gadolinium oxide crystallizing in the Ia-3 crystallographic group, the gadolinium ions occupied the C2 and S6 Wyckoff sites in a 3:1 ratio, alongside europium (III), which served as an active luminescent ion due to the radius similarity [30]. The average crystallite size of Gd_2_O_3_:Eu^3+^ particles, calculated using the Scherrer Equation, using an X-ray wavelength of 0.15418 nm, was 21.4 nm.

**Figure 2:**
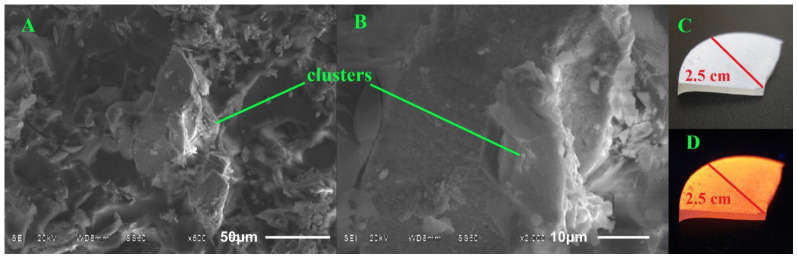
SEM images of the nanoparticle layer on the SiO2–PMMA monolith at (**A**) X500 and (**B**) X2000. Photograph of a sample monolith with radius size around 2.5 cm under (**C**) visible light and (**D**) 250 nm UV light.

During the fabrication, as the gel dried and shrank, Gd_2_O_3_:Eu^3+^ particles precipitated, creating a luminescent layer at the bottom of the container. The interactions between the hybrid gel and the pressure exerted by the shrinking gel compacted this nanoparticle layer into a solid structure supported by the surrounding matrix. Figure 2 shows SEM images of the nanoparticle layer, where it is appreciated that the material is solid and nonporous. No individual nanoparticles can be observed, indicating that the matrix successfully kept the Gd_2_O_3_:Eu^3+^ nanoparticles fixed to the substrate. The layer exhibits cracks and irregularities on its surface, likely caused by tensile forces during the shrinking process of the gel as well as differences in the shrinking rates between the layers with low and high nanoparticle concentrations. Figure 2 also shows photographs (C, D) of a fraction of the sample. The sample is round and 2.5 cm in radius, and the photograph only shows about 1/4 of it due to fracture from handling.

**Figure 3:**
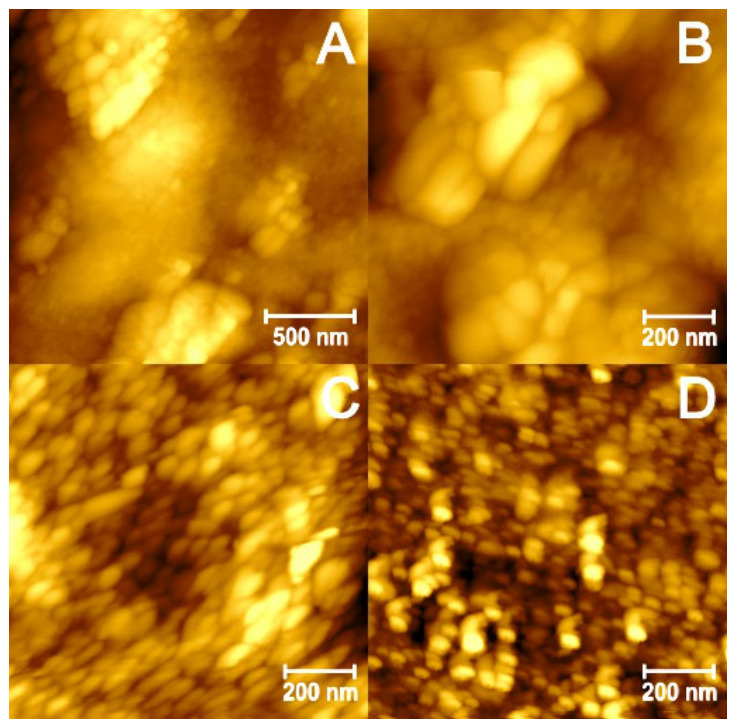
AFM micrographs of the Gd_2_O_3_:Eu^3+^ nanoparticle layer on the SiO_2_–PMMA monolith. (**A**) shows the nanoparticle layer at low magnification, (**B**) centers around a group of clusters, (**C**) focuses on the area between clusters, and (**D**) shows the SiO_2_–PMMA layer.

Images, taken from the low and high-concentration layers using AFM, are presented in Figure 3. In Figure 3A, it can be observed that the surface of the material presents two distinctive zones, prominent protrusions with sharp edges that stand out the surface and a surrounding smoother material. Figure 3B presents an AFM micrograph that focuses on these protrusions, revealing clusters of fused particles with well-defined edges and clear boundaries.

Figure 3C shows an AFM image of the smooth surface surrounding the clusters, highlighting the contrast in smoothness between the areas of protrusions and the protrusions themselves. Figure 3D displays a layer of the SiO_2_–PMMA matrix containing a low concentration of Gd_2_O_3_:Eu^3+^ nanoparticles. The morphologies of the materials in Figure 3C,D are similar, consisting of fused spherical particles. The spherical particles observed in the SiO_2_–PMMA layer are identified as SiO_2_–PMMA particles formed through the sol–gel process. In contrast, the larger clusters are primarily composed of Gd_2_O_3_:Eu^3+^ nanoparticles. Since no other compounds were involved in the synthesis of the material, we propose that the PMMA did not integrate into the sol–gel particles but served instead to fill the spaces between the Gd_2_O_3_:Eu^3+^ particles, providing mechanical support to the lager clusters. The average diameter of Gd_2_O_3_:Eu^3+^ particles was found to be 97 ± 54 nm. This measurement was based on an analysis of 950 particles from the AFM micrographs using ImageJ^®^ version 1.54 g and OriginPro^®^ version 2024 software.

**Figure 4:**
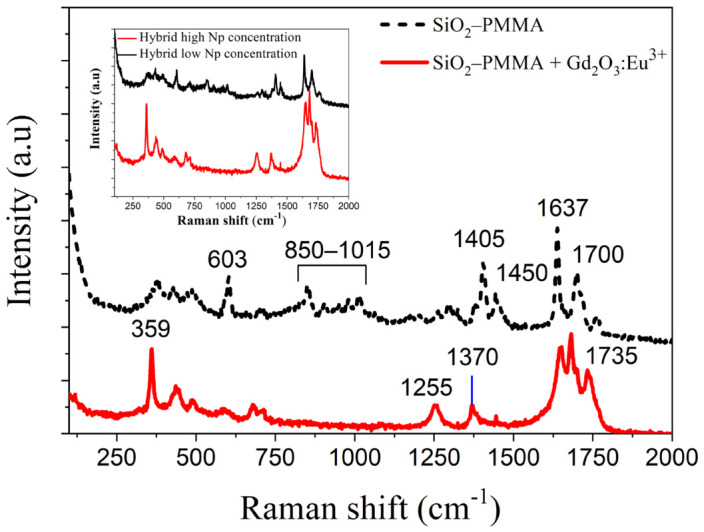
Raman spectra of SiO2–PMMA matrix and SiO2–PMMA + Gd2O3:Eu^3+^ composite. Figure inset: Comparison of Raman spectra between the areas of high and low nanoparticle concentrations in each sample.

Figure 4 presents the Raman spectra of the SiO_2_–PMMA unmodified hybrid and the modified hybrid containing nanoparticles (SiO_2_–PMMA + Gd_2_O_3_:Eu^3+^). The Raman bands identified, as well as their assignments, are shown in Table 1 [31,32,33,34,35,36,37,38]. When Gd_2_O_3_:Eu^3+^ nanoparticles are included, a peak appears at 359 cm^−1^, which corresponds to the F_g_ + A_g_ mode in the cubic phase of the gadolinium oxide, thus confirming the presence of the nanoparticles within the composite. The addition of the Gd_2_O_3_:Eu^3+^ nanoparticles did not generate new peaks, indicating that no chemical bonds were formed between the matrix and the nanoparticles. The spectra revealed changes in the relative intensities of the bands between the doped and undoped samples. The peaks observed at 377, 603, 1013, 1048, 1260, 1410, 1450, 1637, and 1764 cm^−1^ are associated with PMMA chains. Variations in the relative strengths of these peaks indicate a change in the degree of polymerization of PMMA and the overall length of the polymer chains.

An increase in the peaks of 1637, 1700, and 1735 cm^−1^ corresponds to an increase in the number of C=O bonds, due to an overall increase in the degree of polymerization of PMMA and an elongation of the polymer chains. This also explains the reduction in peaks between 1330 and 1450 cm^−1^, which are associated with -CH_3_ and -CH_2_ bonds, the terminal points of the polymer chain.

Changes are observed in Raman bands associated with SiO_2_, specifically in the 433 and 711 cm^−1^ bands, which are assigned to silica rings and Si–C–O bonds, indicating a decrease in the types of bonds. The disappearance of peaks between 850 and 1015 cm^−1^ indicates a reduction in the presence of non-bridging bonds as well as Si–OH bonds. The increase in the 603 cm^−1^ band is attributed to vibrations of the longer polymer chains and the enhanced crosslinking in the SiO_2_ network.

Due to the formation of the two-layer system in the sample, Raman spectra were taken from the high- and low-nanoparticle concentration layers. The inset of Figure 4 presents a comparison of the spectra. It can be observed that the same bands appear in both layers, similar to those in the doped and undoped matrices. The low-concentration layer appears to behave like pure SiO_2_–PMMA, as the characteristic Gd_2_O_3_ peak at 360 cm^−1^ is not detectable. This indicates that this layer contains no measurable amounts of nanoparticles. Additionally, differences in the relative intensities associated with PMMA chains are observed between the low and high nanoparticle concentration layers in the samples. This suggests either a higher amount of polymer or a higher degree of polymerization in the nanoparticle layer. The increased presence of PMMA chains supports the hypothesis that unreacted material from the sol–gel process acts as a binding agent between the nanoparticles.

**Figure 5:**
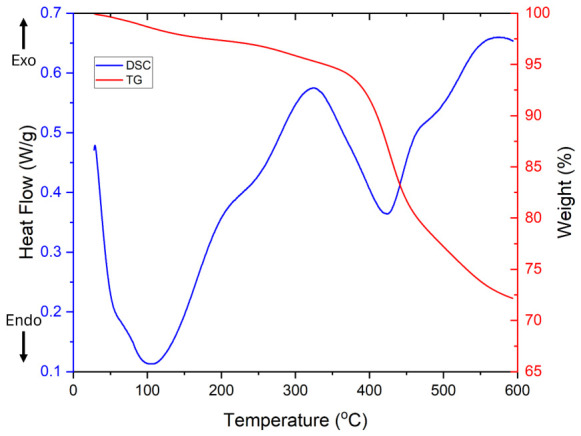
TG and DSC curves of SiO_2_–PMMA matrix with Gd_2_O_3_:Eu^3+^ nanoparticles.

The thermal decomposition during heating of the sample was studied by thermogravimetric analysis (TG) and differential scanning calorimetry (DSC) in a nitrogen atmosphere. The profiles of TG and DSC curves are shown in Figure 5. TG profiles decompose in four main stages: the first three steps correspond to the degradation of PMMA, where a gradual mass loss of 2.2% in the range 28–150 °C can be attributed to the split of the bond between monomers of the head-to-head type; the range 150–280 °C presents a mass loss of 1.5% due to the split at the chain-end initiation from vinylidene ends; and a mass loss of 2.3% in the range of 280–370 °C may be attributed to a random split in the polymer chain [39,40]. The fourth step between the range of 370 and 600 °C represents a mass loss of 22% and relates to degradation of the framework, leading to the formation of metal oxides of Gd, Si, and Eu. The DSC curve shows an endothermic peak centered around 105 °C, which can be associated with the glass transition temperature (Tg) [41], another endothermic peak can be seen around 422 °C, which can be related to the melting temperature (Tm). Finally, the transition towards an exothermic peak around 475 °C can be attributed to decomposition temperature (Td) [42].

**Figure 6:**
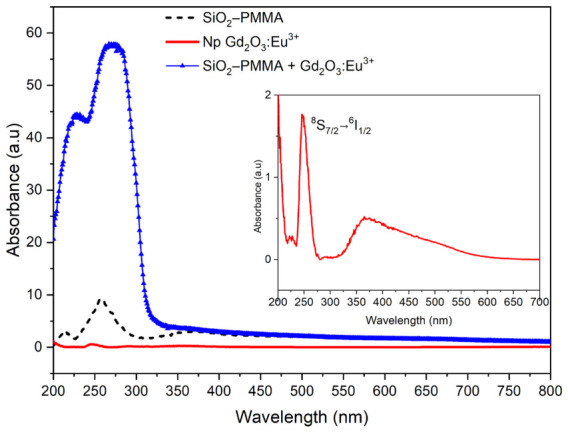
Absorption spectra of the Gd_2_O_3_:Eu^3+^ nanoparticles, SiO_2_–PMMA hybrid material, SiO_2_–PMMA matrix with Gd_2_O_3_:Eu^3+^ nanoparticles (SiO_2_–PMMA + Gd_2_O_3_:Eu^3+^).

The UV–Vis absorption spectra presented in Figure 6 demonstrated that the material exhibited high transparency in the visible region of the electromagnetic spectrum, while showing significant absorption in the UV region. The inset of Figure 6 shows the UV–Vis spectrum of nanoparticles with a peak around 275 nm and can be attributed to a ^8^S_7/2_ → ^6^I_7/2_ Gd^3+^ transition [43]; the presence of the lowest broad band around 365 nm can be explained due to the occupation of Eu^3+^ on Gd^3+^ sites that increase the oxygen vacancies, leading to a change in the electronic structure of the host matrix [44,45]. On the other hand, the hybrid exhibited a similar broader band around 275 nm. This strong absorption in the hybrid material can be attributed to the presence of PMMA chains, which absorb typically in the range between 200 and 400 nm and are known for their high UV absorption capacity [46]. When the nanoparticles are added to SiO_2_–PMMA, the overall absorption of the material is increased across the entire UV spectrum, shown as the blue band of Figure 6. This high level of absorption may hinder the excitation of the luminescent layer through the SiO_2_–PMMA layer. However, although the emitted light can be transmitted through all the composites, this characteristic could be advantageous for specific applications, as it allows for directional control over excitation wavelengths. In contrast, the emitted light is able to pass freely through the material.

**Figure 7:**
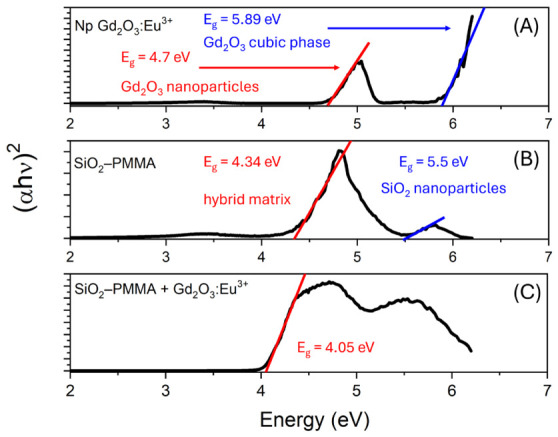
Tauc’s formula (αhν)^2^ vs. photon energy for the SiO_2_–PMMA samples. Gd_2_O_3_:Eu^3+^ nanoparticles (**A**), SiO_2_–PMMA without (**B**) and with (**C**) Gd_2_O_3_:Eu^3+^ nanoparticles.

In Figure 7, the absorption spectra were analyzed to determine the optical bandgap of the samples using Tauc’s method. The direct bandgap of the Gd_2_O_3_:Eu^3+^ nanoparticles shown in section (A) was 5.8 eV, consistent with the cubic phase shown above (Figure 1) and with previous reports on Gd_2_O_3_ materials [1,47]. The obtained bandgap of the SiO_2_–PMMA matrix was 4.34 eV and shown in (B), and the other band at 5.5 eV corresponds to the SiO_2_ nanoparticle gap, which occurs during the sol–gel process in the polymerization of SiO_2_. These nanoparticles are not part of the polymerized SiO_2_ chain and can generally precipitate to the bottom of the sample as it dries. All the deposits that precipitate to the bottom of the sample can be cut and polished to make the sample transparent and homogeneous.

Figure 7C shows the total gap of the sample. When the Gd_2_O_3_:Eu^3+^ nanoparticles were added, the bandgap decrease to 4.05 eV. This reduction in the bandgap may be attributed to the presence Gd_2_O_3_ nanoparticles with the cubic phase in the doped sample, considering that the PMMA bandgap has been reported between 3.6 and 3.9 eV; however, the band gap reduction effect is achieved with the inclusion of metallic nanoparticles, and this is the effect presented here [48,49]. Additionally, previous studies indicated that the bandgap of the SiO_2_–PMMA matrix can vary based on modifications to its composition [50].

**Figure 8:**
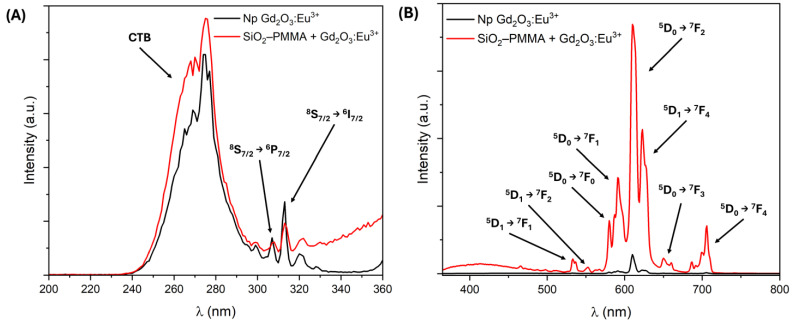
Photoluminescence spectra of Gd_2_O_3_:Eu^3+^ nanoparticles and SiO_2_–PMMA matrix doped with nanoparticles. (**A**) Excitation spectra at emission wavelength 610 nm and (**B**) emission spectra at excitation wavelength 275 nm.

Figure 8A shows the photoluminescence excitation spectra for the Gd_2_O_3_:Eu^3+^ nanoparticles and composite material at an emission wavelength of 610 nm. There is a band centered around 270 nm, which corresponds to the charge transfer band (CTB), the region where the energy transfer between Eu^3+^ ions and O^2−^ from Gd_2_O_3_ takes place. The CTB reaches a maximum at 275 nm regarding Gd ion absorption due to the allowed interconfigurational transition in Gd^3+^ from 4f^7^ (^8^S_7/2_) to 4f^6^ 5d^1^, with the peaks centered at 307 and 314 nm corresponding to the transitions ^8^S_7/2_ → ^6^P_7/2_ and ^8^S_7/2_ → ^6^I_7/2_, respectively; the higher intensity of the peak at 314 nm compared to 307 nm indicates that the Eu ions have more substitution in one of the symmetry sites of the host lattice (C_2_ and S_6_) that enhances the photoluminescent absorption in the host [1,51].Figure 8B shows the photoluminescence emission spectra. The emission from Eu^3+^ ions is evident in the nanoparticles, and this luminescence is retained when the nanoparticles are incorporated into the matrix. The prominent emission peaks occurred at 580, 592, 610, 623, and 706 nm. The highest emission peak is observed at 610 nm, corresponding to the ^5^D_0_ −→ ^7^F_2_ transition of the Eu^3+^ ions.

The peaks at 580 and 592 nm are attributed to the ^5^D_0_ → ^7^F_0_ and ^5^D_0_ → ^7^F_1_ transitions, respectively, while the peaks at 533 and 552 nm are associated with the transitions of ^5^D_1_ → ^7^F_1_ and ^5^D_1_ → ^7^F_2_ [52,53,54]. Additionally, it is noted that the composite exhibits a higher emission intensity compared to the pure Gd_2_O_3_:Eu^3+^ nanoparticles. This increased absorption in the UV range along with the enhanced emission from the doped matrix may be explained by the morphology of the material. The SiO_2_–PMMA matrix separates the Gd_2_O_3_:Eu^3+^ clusters, allowing the incident radiation to penetrate deeper into the material. This separation also facilitates the escape of the emitted light from the composite.

**Figure 9:**
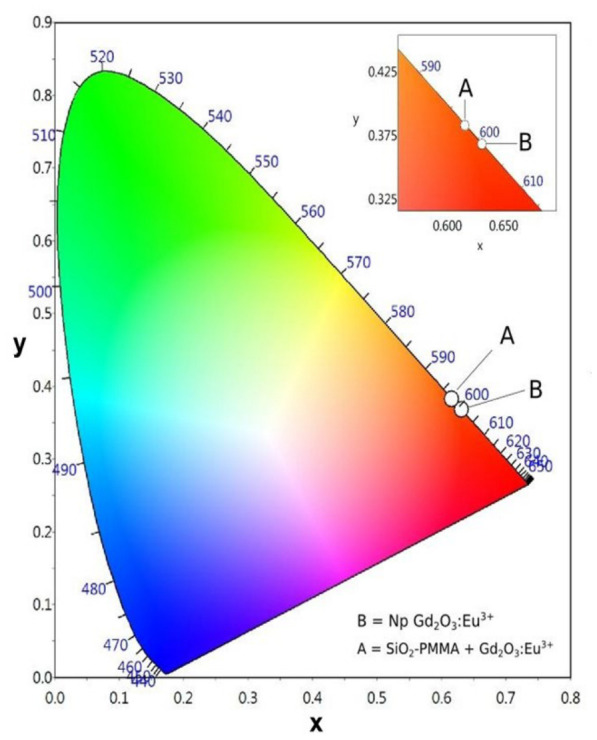
CIE 1931 chromaticity diagram, highlighting their presence inside and outside the matrix for A: Gd_2_O_3_:Eu^3+^ nanoparticles and B: Gd_2_O_3_:Eu^3+^ + SiO_2_–PMMA composite. A zoomed-in section of the graph is shown in the inset.

Figure 9 presents the CIE chromaticity diagram for the emission of the composite, comparing the emission of the Gd_2_O_3_:Eu^3+^ nanoparticles when integrated into the SiO_2_–PMMA hybrid matrix, and shows a slight change in color in the reddish/orange region observed when the nanoparticles are incorporated into the matrix. The material properties mentioned throughout this work are related to the detection of UV radiation, and having the material in the form of a monolith or even in the form of an optical fiber [55] can allow for implementation as waveshifter optical fibers.

The crystalline phase of the matrix dopant is obtained; that is, the cubic phase of Gd_2_O_3_ and nanoparticles of the same element are obtained as part of the original synthesis. The matrix was expected to be amorphous and was synthesized at room temperature. UV–Vis spectroscopy allows for the verification of the presence of formed compounds by calculating the bandgap. In the case of Gd_2_O_3_, the direct transition was used, yielding a result of 4.7 eV for nanoparticles and 5.89 eV for the cubic phase. The indirect transition varies between 5.09 eV and 5.77 eV, meaning there is not much difference if the goal is simply to verify the formation of the compound. The value of the final compound turns out to be more important since the bandgap is around 4.05 eV and it also has an absorption band in the ultraviolet range around 275 nm (Figure 6), which is very important, as shown in Figure 8 of the luminescence spectrum. The absorption spectrum (Figure 6) of the total sample shows the highest spectral value around 275 nm in the ultraviolet region, meaning the matrix absorbs a large amount in this region of the spectrum. This is very important because it helps excite the Eu^3+^ ions, increasing their value, as shown in Figure 8; that is, ion Eu^3+^ transition (black line) is visibly increased in intensity when the ion is inside the matrix, and this is very important for potential applications.

## 3. Conclusions

A SiO_2_–PMMA hybrid material modified with Gd_2_O_3_:Eu^3+^ nanoparticles was synthesized using the sol–gel technique. The nanoparticles were successfully integrated into the SiO_2_–PMMA matrix without losing their properties, resulting in a luminescent composite. However, a homogeneous solid could not be obtained because most of the nanoparticles precipitated during the drying process of the samples. Instead, the nanoparticles formed a high concentration layer, held together by the SiO_2_–PMMA material, while the remainder of the matrix contained a lower concentration of nanoparticles. Although the composite did not behave as initially intended, other beneficial effects were observed that could be useful for multiple applications. Our results indicate that the nanoparticles formed luminescent clusters within the transparent matrix, which enhanced some of the optical properties of the composite. Additionally, the nanoparticles exposed to the ambient atmosphere exhibited high luminescence, a characteristic that could be advantageous in developing sensors. The thickness of the layers could also be easily controlled by adjusting the ratio between the matrix and the nanoparticles. Nonetheless, more research is needed to understand the interactions between the SiO_2_–PMMA matrix gel and the Gd_2_O_3_:Eu^3+^ nanoparticles, as a similar phenomenon could be leveraged to design other innovative materials. We have a solid material based on the formation of a gel, the polymerization of a monomer, and the inclusion of nanoparticles and Lanthanide ions whose physical dimensions can vary to the point of using a mold like polymers and obtaining tablets, fibers, and large monoliths of the required geometric shapes. The material matrix absorbs a wide range (between 200 and 350 nm) of the ultraviolet region, and the highest emission band is around 610 nm (red color); so, we have a material for molding: waveshifter fibers, a waveguide for optics, and a scintillator plastic detector of high energy radiation, such as α and β particles and even cosmic rays.

## 4. Materials and Methods

To synthesize nanoparticles, europium (III) nitrate pentahydrate (Eu(NO_3_)_3_·5H_2_O, 99%), gadolinium (III) nitrate hexahydrate (Gd(NO_3_)_3_·6H_2_O, 99%), and ammonium hydroxide (NH_4_OH) were used, with polyvinyl pyrrolidone ((C_6_H_9_NO)_n_, 99%) serving as the surfactant. In the case of SiO_2_–PMMA hybrid material synthesis, tetraethyl orthosilicate (SiC_8_H_20_O_4_, 98%, TEOS), methyl methacrylate (C_5_H_8_O_2_, MMA, 99%), and 3-(Trimethoxysilyl) propyl methacrylate (C_10_H_20_O_5_Si, TMSPM, 98%) were used. All reagents were purchased from Sigma-Aldrich and used without further purification. Commercial medical-grade distilled water was used for hydrolysis, and ethanol (99.9%, J.T. Baker) acted as the solvent. Benzoyl peroxide (C_14_H_10_O_4_, 98%, Sigma Aldrich) was employed as the catalyst, while sodium hydroxide pellets (NaOH, 98%, J.T. Baker) were used to control the pH of the solution.

The Gd_2_O_3_:Eu^3+^ nanoparticles were synthesized through a precipitation reaction. Initially, 0.5 g of Gadolinium (III) nitrate hexahydrate (Gd(NO_3_)_3_·6H_2_O) and 0.01 g of Europium(III) nitrate pentahydrate (Eu(NO_3_)_3_·5H_2_O) were dissolved in 20 mL of water and stirred for 30 min to obtain a homogeneous mixture. Next, 0.5 mL of ammonium hydroxide (NH_4_OH) was added drop by drop to the mixture. As the reaction progressed, Gd(OH)_3_ particles were formed as a white precipitate. Then, this precipitate was centrifuged and washed at least three times with distilled water. The product was dried in an oven at 80 °C and treated at 600 °C for 4 h to obtain Gd_2_O_3_ particles via the thermal decomposition of the precursors.

The SiO_2_–PMMA hybrid was synthesized using the sol–gel technique, which involved the simultaneous hydrolysis of TEOS and the polymerization of MMA in an ethanol solution. TMSPM served as bonding agent between the SiO_2_ and PMMA molecules. The molar proportions used were TEOS:MMA:TMSPM:H_2_O:ethanol in a ratio of 1:1:0.22:4.75:4.75. Benzoyl peroxide was added as a catalyst for the MMA polymerization at a 1% concentration relative to MMA. NaOH was incorporated into the solution to regulate the acidity; an optimal pH between 9 and 10 was determined for the gelation of the sol. To modify the matrix, 20 mg of europium-doped Gd_2_O_3_ nanoparticles were incorporated into 10 mL of the hybrid sol. The sol was then allowed to gel and dry in a sealed container, which had a small opening to facilitate slow and continuous solvent evaporation.

The weights, volumes, and concentrations employed for the synthesis were chosen according to the suitable incorporation of the solutions in each stage. For the synthesis of Gd_2_O_3_:Eu^3+^ nanoparticles, a range of concentrations from 1 to 3 wt% of Eu^3+^ ions was tested. The SiO_2_–PMMA hybrid has a hydrolysis and condensation stage in which the addition of H_2_O must be drop by drop, and also the NaOH must be added slowly and drop by drop to avoid an uncontrolled gelation and further opacification of the material. The value of the pH was tested from 6 to 10, finding the optimum between 9 and 10. The modification of the matrix with the addition of the phosphor was studied in a range of concentrations of 1 to 3 mg/mL. The optimum nanoparticle load was chosen regarding the monolith formation; at low concentrations, the light emission characteristic of the Eu^3+^ ions is not recognizable, and at higher concentrations, the solution reaches a saturation leading to the opacification of the monolith.

The samples were characterized by X-Ray diffraction using the equipment Panalytical Empyrean diffractometer (London, England), using the Bragg–Brentano configuration and a Cu-Kα (1.5406 Å) radiation source with a pass time of 45 s. The surface of the samples was analyzed using a JEOL Scanning Electron Microscope model JSM-5600LV (Tokyo, Japan) at WD 8 mm and 20 kV. Raman spectroscopy (LabRAM, Horiba Scientific, Paris Saclay, France), with excitation from a HeNe laser at a wavelength of 633 nm from 200–2000 cm^−1^, was used to study the chemical composition of the samples. Utilizing thermogravimetric (TG) and differential scanning calorimetry (DSC) methods, using a DSC–TGA equipment brand Waters model Discovery SDT 650 (Massachusetts, USA), a sample (about 31.3 mg) was heated from 28 to 600 °C at a rate of 10.0 °C·min^−1^ under a nitrogen atmosphere. A spectrofluorometer Nanolog Fluorescence model FR3 (Horiba Jobin Yvon, Kyoto, Japan), was utilized to characterize the excitation spectra using an emission wavelength of 610 nm, as well as the emission spectra of the samples using an excitation wavelength of 275 nm. The UV–Vis absorption spectra were measured with a Spectrophotometer UV–Vis–NIR Cary 5000 (Varian–Agilent, Santa Clara California, USA) through diffuse reflectance. AFM (XE7, Park Systems, Gwacheon, Korea) micrographs were taken using a tapping mode and a C-soft tapping cantilever (Budget Sensors, Tsarigradsko Shose, Bulgaria).

## Figures and Tables

**Table 1:** Raman bands and their assignments of samples. The assignments match with references [31,32,33,34,35,36,37,38] in the last column.

Frequency (cm^−1^)	Assignment	Reference
359	F_g_ + A_g_	[31]
377	CC^a^C	[36]
433	≥5 fold-ring (ω_1_)	[32]
488	D_1_	[32]
603	D_2_, ν(C-COO), ν_s_(C-C)	[32,33]
661	Si-O-C	[33]
710	Si-O-C	[35]
854	Si-O-2NBO stret.	[32,33]
904	Si-O-2NBO stret.	[32]
980	Si-OH sym stret.	[32]
1015	-CH_3_	[33,38]
1048	ν(C-C)	[33,38]
1265	ν(C-C), ν(C-COO)	[33]
1300	Si-CH_3_	[34]
1328	-CH_2_	[34,36]
1370	α-CH_3_	[34,36]
1405	-CH_3_	[34,36]
1450	δ_a_(C-H) of α-CH_3,_ δ_a_(C-H) of O-CH_3_	[33,36]
1637	O-H, ν(C=C), ν(C-COO)	[33,34,37]
1700	ν(C=O)	[33,37,38]
1735	ν(C=O)	[33,36,38]

## Data Availability

Data is contained within the article.
